# The Influence of Hand Preference on Grip Strength in Children and Adolescents; A Cross-Sectional Study of 2284 Children and Adolescents

**DOI:** 10.1371/journal.pone.0143476

**Published:** 2015-11-23

**Authors:** Ann M. Hepping, Joris J. W. Ploegmakers, Jan H. B. Geertzen, Sjoerd K. Bulstra, Martin Stevens

**Affiliations:** 1 Department of Rehabilitation Medicine, University Medical Center Groningen, University of Groningen, Groningen, the Netherlands; 2 Department of Orthopedics, University Medical Center Groningen, University of Groningen, Groningen, the Netherlands; University of Ottawa, CANADA

## Abstract

**Introduction:**

In adults the preferred hand is often considered to be around 10% stronger than the non-preferred hand. Whether the same is true for children and adolescents remains unclear. The objective of this study is therefore to determine whether there is a difference in grip strength between the preferred and non-preferred hand in developing children, to establish whether this difference is similar for children of a different gender or hand preference, and whether there is a difference in grip strength of the preferred hand of left-preferent (LP) and right-preferent (RP) children.

**Design:**

Cross-sectional study.

**Participants:**

Participants were recruited from schools in the northern provinces of the Netherlands. The study included healthy children and adolescents in the age range of 4–17 years.

**Outcome Measures:**

Each child was allowed a total of four attempts using the JAMAR hand dynamometer, two attempts with each hand. All individual attempts were scored. Hand preference was determined by asking which hand was used to write, or in the case of 4- and 5-year-olds, which hand was used to draw a shape.

**Results:**

The study population comprised 2284 children and adolescents. RP boys and girls scored significantly higher with their preferred hand, the difference amounting to 9.5 and 10.1% respectively. LP girls scored significantly higher with their preferred hand, but this difference was only 3.0%. For LP boys no significant difference was found in favor of either hand. LP children score higher with the non-preferred hand and tie scores on both hands more often than RP children.

**Conclusion:**

The 10% rule of hand preference is applicable to RP children ranging in age between 4 and 17 years, but not to LP children. In contrast to LP boys, LP girls are generally significantly stronger with their preferred hand.

## Introduction

Grip strength measurements have a profound role as a parameter that is reflective of hand function. For this reason, they are used in the evaluation of patients with a large variety of pathologies. When assessing actual degree of impairment, for example in terms of recovery after trauma or surgery, patient values are often compared with reference values. Unfortunately, reliable reference values from a representative study population are not always available, and moreover they do not take into account individual personal characteristics that determine strength. Comparison of the values of the affected and the unaffected hand of the same patient thus provides an alternative method to estimate level of impairment. As such, it is recommended by the American Association of Hand Therapists and the American Medical Association [1–3]. However, when making comparisons with the unaffected side, the question arises as to whether hand preference is a factor that must be taken into account.

In 1954 a study performed by Bechtol concluded that the dominant hand was, on average, 5–10% stronger than the non-dominant hand [4]. Since then, many studies have examined more thoroughly the influence of hand dominance, or preference, on strength in adults, thereby challenging this statement–often referred to as the 10% rule [5]. Results from the various studies on this topic are far from conclusive though [6,7]. While some studies found that hand dominance had no important influence on grip strength [8–10], others identified significant, albeit more subtle, differences [11], and yet other studies concurred with the 10% rule but only for specific groups [5,12–15]. Such inconsistencies are probably the result of the varying methods and inclusion criteria between studies, as well as a relatively small number of left-preferent individuals tested. When it comes to the grip strength of children, much less research has been performed in general. A search of the literature was unable to identify any studies that examined if or how the influence of hand preference on grip strength develops from childhood to adolescence, or whether there are any differences in this influence between boys and girls or between children with a different hand preference. We therefore believe that there is no clear answer as to how hand preference affects grip strength in children and adolescents.

This study aims to establish the influence of hand preference on grip strength in more detail by challenging the 10% rule in both left-preferent (LP) and right-preferent (RP) boys and girls aged 4–17, as well as to compare the absolute scores between children with a different hand preference. The research questions thus were:

1. Is there a difference between the grip strength of the preferred hand and the non-preferred hand in children?
2. If so, is this difference similar for children with another hand preference?
3. Is there a difference in grip strength between the preferred hands of LP versus RP children?

## Methods

### Study design and participants

This study is part of a large cross-sectional study determining reference values of grip strength of children in the Netherlands [16]. The Medical Ethical Board of University Medical Center Groningen specifically approved the consent procedure of this study (M13.142928). Healthy children age 4–17 were included by approaching schools in the four northern provinces of the Netherlands. Exclusion criteria comprised pain or restriction of the arm or hand at the time of examination, conditions interfering with normal growth, neuromuscular and generalized bone diseases, and inability to use the dynamometer as instructed. Parents of the children were informed about the study by means of a letter. If parents had objections regarding their child’s participation, the child was not enrolled. Permission of the children was obtained verbally. A list of all children was provided by the teacher of the class. On this list it was registered which parents had objections regarding participation and in addition which child agreed and which child refused to participate. We made sure that the child knew the examination was not mandatory, and children were not included if they didn’t want to participate themselves. Data were processed anonymous. The protocol of the study was approved by the Medical Ethical Board of University Medical Center Groningen (M13.142928).

### Outcome measures

Researchers gave a short introduction at the start of each measurement session to explain the purpose and procedures of the study. Use of the dynamometer was demonstrated by letting the teacher perform a grip strength measurement. All measurements took place in a private room at the child’s school. Due to the large number of children that needed to be included, medical students aided performing the measurements, under direct supervision of one of the two researchers (AMH, JJWP). Children were allotted to the respective age groups based on their calendar age at the time of examination. For example, a child was considered to be a 4-year-old from the day of its 4^th^ birthday up to the day before its 5^th^ birthday. To establish hand preference, children were asked what hand they use to write, or in case of 4- and 5-year-olds which hand was used to perform other activities such as cutting or drawing. As an additional confirmation, 4- and 5-year-olds as well as older children who displayed uncertainty about the answer were asked to draw a shape. To this end, they were asked to pick up a pen from the table themselves, to avoid possible bias from the researcher. The hand that was used to draw was then scored to be the preferred one.

### Measurements

To measure grip strength the Jamar^®^ hydraulic hand dynamometer (JHD) (Lafayette Instrument Company, Lafayette, IN, USA) was used. Subjects were assessed according to the standardized testing position as advised by the American Society of Hand Therapists (ASTH): seated subject, shoulders adducted and neutrally rotated, elbow flexed at 90°, wrist between 0 and 30° extension and between 0 and 15° ulnar variation [17,18]. For all 4- and 5-year-olds the handle of the device was set to the first position and they were allowed to manually support the tested arm with the contralateral hand. For all other subjects the handlebar was set to the second position and supporting the tested arm was prohibited. All subjects were allowed two attempts with each hand with a 10-second break between measurements, and the starting hand was alternated between subjects. Verbal encouragement was given and it was attempted to keep tone and volume as consistent as possible. A Dutch translation of the Southampton Grip Strength Measurement Protocol was used: counting down from 3 to 0, followed by “squeeze as hard as you can … squeeze and let go” [19].

### Data analysis

Descriptive statistics for the main characteristics of the study population were tabulated. To answer all research questions, several two-level multilevel analyses were performed with the mean grip score of the left and right hand as dependent variable, nested under the children as second level. The first model is the empty model to estimate intraclass correlation. Age, gender and hand preference as characteristics of the children and the hand measured (left or right) were included as fixed factors. From the empty model three models were used to answer the three research questions in the total group, thereafter adjusted for gender and subsequently for gender and age [20,21]. Results were regarded as significant if the associated p-value was < 0.05. Statistical procedures were carried out using SPSS 22.0 for Windows (IBM SPSS Inc.).

## Results

The total study population comprised 2284 children, of whom 1980 were (RP) and 304 (LP). Overall, 15.9% of boys preferred their left hand versus 10.7% of girls. A detailed overview of the study population and the results of grip strength measurements can be found in Table 1. Unfortunately there was a decline in the number of participants aged 14 and older included in the study, so for statistical purposes these children were analyzed as a single subgroup (age group 14+) to provide a larger sample size.

**Table 1:** Overview of results: mean grip strength (kg) of both hands according to age, gender and hand preference.

	Boys						Girls					
	Right-preferent			Left-preferent			Right-preferent			Left-preferent		
Age	N (%)	Left hand	Right hand	N (%)	Left hand	Right hand	N (%)	Left hand	Right hand	N (%)	Left hand	Right hand
**4**	91 (73.4%)	5.1 (2.1) 1.5–10.0	5.8 (2.2) 2.0–11.5	33 (26.6%)	5.3 (2.3) 1.0–10.0	5.6 (2.4) 2.0–10.0	96 (88.1%)	4.5 (2.0) 1.5–9.5	4.9 (2.1) 1.0–11.0	13 (11.9%)	6.2 (2.1) 2.5–9.5	6.0 (1.8) 2.5–8.0
**5**	84 (82.4%)	6.8 (2.6) 2.5–12.5	7.7 (2.5) 3.0–13.0	18 (17.6%)	6.5 (2.9) 2.0–14.0	6.8 (2.6) 3.0–13.5	95 (90.5%)	6.0 (2.4) 1.0–12.0	6.8 (2.3) 2.0–14.5	10 (9.5%)	6.3 (3.1) 2.0–11.5	6.0 (2.9) 1.5–10.5
**6**	102 (82.9%)	9.3 (2.7) 4.0–16.5	10.1 (2.6) 5.0–17.5	21 (17.1%)	10.5 (2.5) 7.5–16.5	10.1 (2.8) 6.5–16	93 (86.1%)	8.4 (3.1) 2.0–15.5	9.2 (2.8) 3.0–18.0	15 (13.9%)	8.1 (2.3) 5.0–13.0	7.9 (3.3) 2.5–14.0
**7**	89 (85.6%)	11.8 (3.4) 4.5–19.0	13.1 (3.3) 7.5–21.0	15 (14.4%)	12.5 (3.2) 6.5–16.5	12.7 (3.4) 8.0–17.5	91 (92.9%)	11.8 (2.9) 5.0–17.5	12.9 (2.9) 6.5–20.5	7 (7.1%)	13.4 (3.8) 9.0–18.0	12.9 (2.8) 10.0–16.0
**8**	100 (88.5%)	14.7 (3.2) 8.0–23.0	16.1 (3.4) 8.0–25.0	13 (11.5%)	14.4 (4.4) 8.0–22.0	14.3 (3.8) 8.0–22.0	107 (90.7%)	13.0 (2.8) 6.5–20.5	14.4 (2.9) 8.0–22.0	11 (9.3%)	14.2 (2.5) 10.5–18.0	14.0 (1.5) 12.0–16.0
**9**	103 (88.8%)	16.7 (3.8) 7.5–32.5	18.3 (3.7) 10.0–29.0	13 (11.2%)	17.2 (2.9) 12.5–23.0	17.9 (4.1) 12.0–25.0	105 (88.2%)	15.1 (3.0) 7.0–23.0	16.8 (3.2) 9.0–25.5	14 (11.8%)	15.6 (3.2) 10.5–21.0	14.8 (3.3) 10.0–21.0
**10**	88 (80.7%)	17.7 (3.2) 9.0–27.5	19.5 (3.6) 11.5–28.5	21 (19.3%)	19.7 (2.8) 13.5–25.0	19.6 (3.4) 12.0–24.5	88 (85.4%)	16.9 (3.9) 7.5–28.5	19.1 (3.6) 9.0–29.0	15 (14.6%)	19.1 (3.7) 13.0–26.5	18.6 (3.7) 10.5–25.5
**11**	96 (85.0%)	20.7 (4.5) 7.5–33.0	22.5 (4.9) 9.0–34.5	17(15.0%)	19.6 (4.3) 11.5–28.0	19.9 (4.3) 11.0–28.0	98 (86.7%)	18.8 (3.9) 11.0–30.0	20.8 (4.1) 10.0–34.5	15 (13.3%)	21.6 (3.9) 15.5–30.0	20.9 (3.8) 16.5–30.0
**12**	80 (83.3%)	23.0 (4.5) 13.0–35.0	25.1 (4.8) 14.5–36.5	16 16.7%)	23.0 (4.4) 13.0–31.5	22.7 (4.8) 15.0–32.0)	97 (91.5%)	22.3 (4.4) 13.0–33.0	24.4 (4.8) 15.0–38.5)	9 (8.5%)	21.7 (3.0) 18.0–26.5	22.3 (3.8) 16.0–30.0
**13**	61 (92.4%)	26.1 (6.1) 16.5–41.5	28.4 (6.3) 17.0–45.0	5 (7.6%)	25.8 (5.6) 20.0–33.0	22.1 (4.1) 17.0–28.5	92 (94.8%)	24.6 (4.4) 16.5–36.0	26.5 (4.7) 18.0–38.5	5 (5.2%)	23.0 (6.0) 13.5–30.0	23.1 (5.6) 17.5–31.0
**14+**	56 87.5%)	34,6 (7.8) 22.0–57.0	37,5 (7.7) 24.0–62.0	8 (12.5%)	26,1 (5.4) 13.0–39.0	28.8 (5.2) 16.0–39.0	68 (87.2%)	35.8 (7.7) 25.5–48.0	37.3 (6.3) 28.0–47.0	10 (12.8%)	33.1 (5.0) 26.5–43.0	30.9 (4.0) 24.5–36.0
**Total**	950 (84.1%)			180 (15.9%)			1030 (89.3%)			124 (10.7%)		

Data for each hand is presented in kg as: Mean (SD), minimum–maximum.

### Difference between grip strength of the preferred and non-preferred hand

The grip strength of the preferred hand was first compared to that of the non-preferred hand. This showed that the preferred hand was significantly stronger (p<0.001) for the study population as a whole. Further analysis showed that the same holds true for boys and girls tested separately (p<0.001) as well as for all the different age groups. Results can be found in Table 2 (section A).

**Table 2:** Results of comparisons of: A. Score of the preferent versus score of the non-preferent hand. B. Score of the preferent versus score of the non-preferent hand in LP and RP children tested separately. Analyses been performed on group level, according to gender, and finally according to gender and age.

	A			B					
	Preferred versus non-preferred			Left-preferent			Right-preferent		
	Mean Difference	SE*	P-value	Mean Difference	SE*	P-value	Mean Difference	SE*	P-value
**Total Group**	1.330	0.046	<0.001	0.166	0.123	0.176	1.509	0.048	<0.001
**Boys**	1.262	0.065	<0.001	-0.047	0.159	0.767	1.510	0.069	<0.001
**Girls**	1.397	0.064	<0.001	0.476	0.192	0.013	1.508	0.067	<0.001
**Boys 4 yrs**	0.391	0.190	0.039	-0.242	0.358	0.498	0.621	0.216	0.004
**Boys 5 yrs**	0.716	0.209	0.001	-0.361	0.485	0.456	0.946	0.224	<0.001
**Boys 6 yrs**	0.744	0.191	<0.001	0.405	0.449	0.367	0.814	0.204	<0.001
**Boys7 yrs**	1.024	0.207	<0.001	-0.200	0.531	0.707	1.230	0.218	<0.001
**Boys 8 yrs**	1.257	0.199	<0.001	0.154	0.570	0.787	1.400	0.206	<0.001
**Boys 9 yrs**	1.371	0.196	<0.001	-0.692	0.570	0.225	1.631	0.203	<0.001
**Boys 10 yrs**	1.472	0.202	<0.001	0.071	0.449	0.874	1.807	0.219	<0.001
**Boys 11 yrs**	1.460	0.199	<0.001	-0.324	0.499	0.517	1.776	0.210	<0.001
**Boys 12 yrs**	1.786	0.216	<0.001	0.344	0.514	0.504	2.075	0.230	<0.001
**Boys 13 yrs**	2.356	0.260	<0.001	3.700	0.920	<0.001	2.246	0.263	<0.001
**Boys 14** ^**+**^ **yrs**	2.391	0.264	<0.001	-1.562	0.727	0.032	2.955	0.275	<0.001
**Girls 4 yrs**	0.406	0.202	0.045	0.231	0.570	0.686	0.430	0.210	0.041
**Girls 5 yrs**	0.719	0.206	0.001	0.300	0.650	0.645	0.763	0.211	<0.001
**Girls 6 yrs**	0.681	0.203	0.001	0.167	0.531	0.754	0.763	0.213	<0.001
**Girls 7 yrs**	1.005	0.214	<0.001	0.429	0.777	0.582	1.049	0.216	<0.001
**Girls 8 yrs**	1.212	0.195	<0.001	0.136	0.620	0.826	1.322	0.199	<0.001
**Girls 9 yrs**	1.601	0.194	<0.001	0.786	0.550	0.153	1.710	0.201	<0.001
**Girls 10 yrs**	1.913	0.208	<0.001	0.567	0.531	0.286	2.142	0.219	<0.001
**Girls 11 yrs**	1.845	0.199	<0.001	0.733	0.531	0.167	2.015	0.208	<0.001
**Girls12 yrs**	1.910	0.205	<0.001	-0.667	0.686	0.331	2.149	0.209	<0.001
**Girls 13 yrs**	1.840	0.215	<0.001	-0.100	0.920	0.913	1.946	0.214	<0.001
**Girls 14** ^**+**^ ** yrs**	2.571	0.239	<0.001	2.200	0.650	0.001	2.625	0.249	<0.001

*Se = standard error.

### Difference between grip strength of the preferred and non-preferred hand according to hand preference

Next, we analyzed whether this difference in strength in favor of the preferred hand exists for left- as well as RP children. RP children are significantly stronger with their preferred hand (p<0.001). Again, the same is true when boys (p<0.001) and girls (p<0.001) are analyzed separately, as well as for all the individual age groups (see Table 2 (section B)). In terms of percentage, the advantage of the preferred hand was similar for both genders and relatively stable across the age groups. RP boys scored 9.5% higher on the average of two grip strength measurements with their preferred hand, fluctuating from 8.5–13.9% between the respective age groups. For RP girls this amounted to 10.1%, fluctuating from 7.9–12.7%.

By contrast, among LP children no difference in favor of either hand was found (p = 0.176). Similarly, when dividing the LP group according to gender no significant difference for boys was found (p = 0.767); overall, LP boys scored 0.4% lower with their preferred hand. LP girls were significantly stronger with their preferred hand (p = 0.013), but the benefit of hand preference on strength was less evident compared to RP girls, namely 3.0% higher. The results according to the separate age groups are presented in Table 2 (section B).

**Fig 1:**
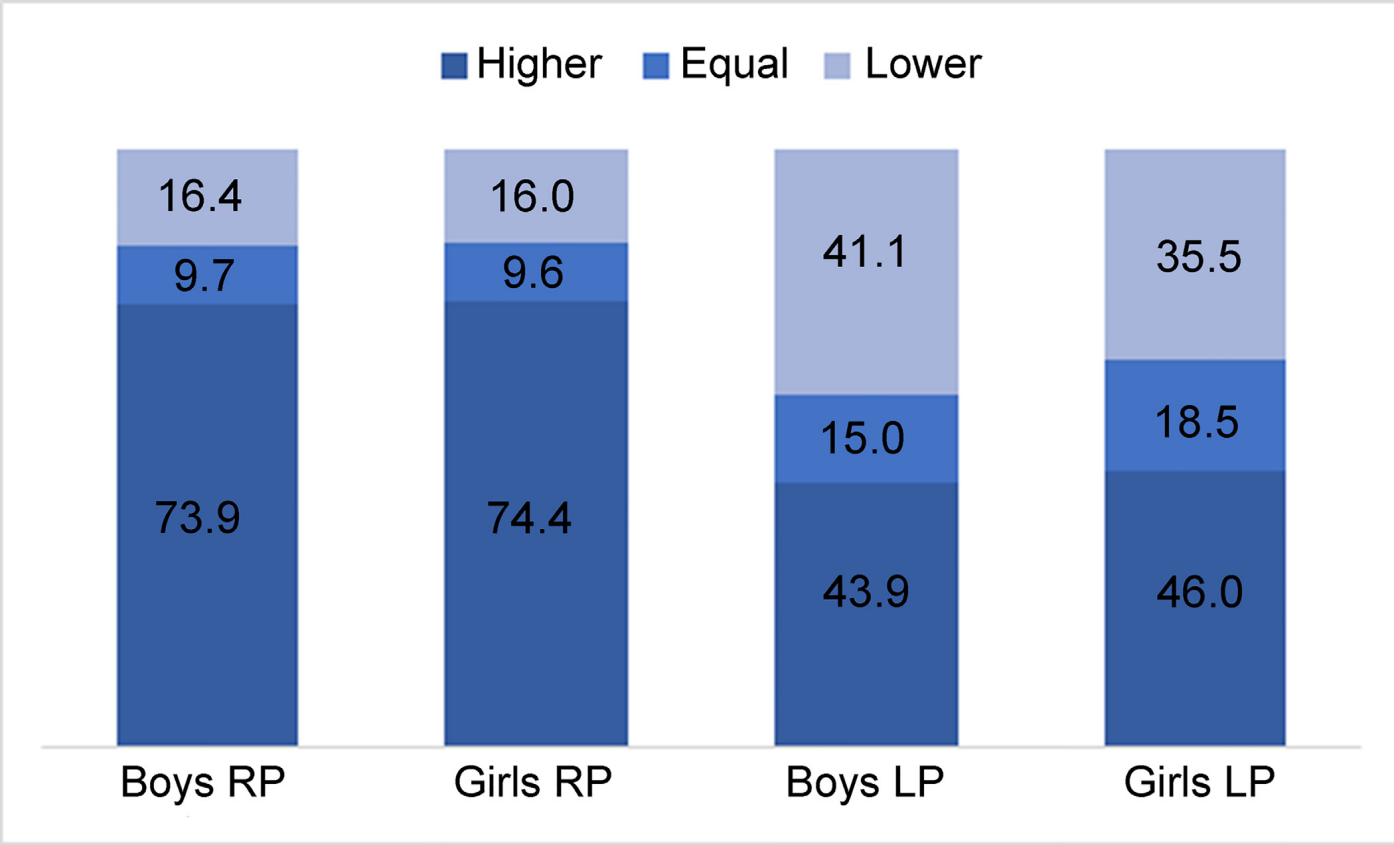
Percentage of children that scored higher, equal, or lower with their preferred hand compared with their non-preferred hand, according to hand preference and gender.

When assessing the differences in grip strength between left- and RP children from a different point of view, the children were divided into groups that scored higher, equal or lower with their preferred hand compared to their non-preferred hand, as represented in Fig 1. As can be seen, 16% of RP children scored higher with their non-preferred hand, and around 10% of children tied scores; these results were consistent for boys and girls. LP children scored higher with the non-preferred hand more often, at 36% for girls and 41% for boys. Scoring equally with both hands was also more frequent, at 15% and 19% of LP girls and boys respectively.

### Difference in grip strength between left- and RP children

Lastly, the grip strength of the preferred hand of LP children was compared to that of RP children; similarly this was done for the non-preferred hand. For the preferred hand this showed a significant difference (p = 0.001) in favor of the RP group. The same was true for boys tested separately (p<0.001) but not for girls (p = 0.486). For the non-preferred hand, no significant difference in strength was found in favor of either RP or LP children (p = 0.583), nor for boys (0.079) or girls separately (0.454). Since the results according to gender were non-significant in 3 of the 4 groups tested, results according to the even smaller age groups are not further discussed here. Results of the total analysis have been tabulated for reasons of consistency as well as to facilitate possible future data comparison and can be found in the S1 Appendix.

## Discussion

The results show that the 10% rule of the dominant hand regarding grip strength in adults holds true for RP children as a group, as well as for boys and girls of all age groups tested separately. However, the 10% rule cannot be generalized for LP children. LP girls are significantly stronger with their preferred hand as a group, but this effect is less evident, amounting to 3%, and thus not present in all of the separate age groups. For LP boys no significant difference in strength in favor of either hand was found. These findings should be taken into account when grip strength measurements are used to assess the degree of impairment or to monitor the patients recovery, as is often done by hand therapists. While the vast majority of RP children scored higher with their preferred hand compared with their non-preferred hand, the same does not hold true for LP children. It is much more common for LP children to have equal scores, or even score higher with their non-preferred hand. This might also contribute to the score of the preferred hand of RP children being significantly greater than that of LP children. The latter was only true for the entire group and could not be observed as a clear trend when the age groups where analyzed separately.

As with studies on grip strength measurements in adults, studies that focus specifically on children have come to different conclusions regarding the influence of hand preference. The current study’s results are in agreement with findings of De Smet and Vercammen (2001); they stated that the 10% rule did hold true for right-hand dominant children, yet their results yielded a non-significant difference. They also found no difference between the strength of hands in left-hand dominant children, but did not look at boys and girls separately [13]. Ager et al. (1984) and Bear-Lehman et al. (2002) stated that hand dominance was not significant [8,22]. Molenaar et al. (2010) did not evaluate children with a different hand preference separately, and Newman et al. (1984) did not assess results as relative percentages, therefore adequate comparison was not possible [23,24]. Several studies focusing on adults have described that the 10% rule only holds true for right-dominant individuals and that no difference for left-hand-dominant individuals could be found [5,12–15].

Regarding the percentage of children that scored higher, equal or lower with their preferred hand compared to their non-preferred hand according to hand preference, a similar phenomenon has also been described in adults. Incel et al. (2002) illustrated in their study that left-hand-dominant adults are frequently stronger with their non-dominant hand compared with their dominant hand as right-hand dominant adults, namely 33.3% versus 10.9% [14]. Petersen et al. (1989) described the same, but found a much larger difference of 48% versus 6.9% [5]. A search of the literature did not identify any studies examining this in children. The current findings closely resemble those of Incel et al. (2002), which might suggest that these differences remain relatively stable from childhood into adulthood [14].

Finally, regarding to the comparison of the strength of LP versus that of RP children, Mathiowetz et al. (1986) evaluated the interpersonal scores of both hands and found no differences [9]. However, they compared the scores of the left and right hands between children of a different hand-preference, whilst we compared the scores of the preferred and non-preferred hands. Current results did show a significant difference for the score of the preferred hand on group level as well as for boys.

The absence of a significant difference in strength in favor of the preferred hand in LP individuals has been described previously. Often this was considered to be a consequence of social pressures to become right-handed, but it is an unlikely explanation for the current differences in the Netherlands [25]. A Dutch study conducted in 1985 reported that the percentage of left-dominant individuals that actually wrote with their left hand rose from 0% for persons born between 1910 and 1939 to 100% for those born after 1965 [26], suggesting that the writing hand can be considered to be the preferred or dominant one. This would concur with more recent studies stating that the hand used to write is the most important predictor of hand dominance in children, more so than performance of other activities, whereas the same did not hold true for adults at the time [27,28]. An alternative theory would be that LP children are still forced to become ambidextrous in modern times because objects that are used in daily living are often specifically designed for right-handed individuals. This however would not explain why such differences are already present at the age of 4 and remain relatively consistent across different age groups. Moreover, it does not explain why LP girls are significantly stronger with their preferred hand whilst LP boys are not. The differences in grip strength of the preferred hand and non-preferred hand between LP and RP children are therefore likely to have an intrinsic basis rather than a solely environmental one.

The degree to which left-hand-dominant individuals use their dominant hand is known to be lower than that of right-hand-dominant individuals. Similar differences between left- and right-dominant individuals are reported in studies focusing on accuracy and speed, and on a higher level in motor-evoked potentials by transcranial magnetic stimulation [29,30]. A review by Scharoun et al. (2014) showed that in studies assessing hand preference by means of questionnaires, young left-dominant individuals demonstrated weak preferences while their right-dominant counterparts reported a consistent preference from a young age [31]. This difference is likely to contribute to the variations in grip strength between LP and RP children and to be a consequence of nature instead of nurture, especially at a young age (3–5 years). Several studies in adults have shown that there are differences regarding the activation of the motor cortex between LP and RP individuals. Vingerhoets et al. (2012) concluded that whilst pantomiming tool movements the left lateralized activation pattern is similar for LP and RP individuals, that LP individuals show less asymmetry, and that hand preference does not influence the side of lateralization but only the strength [32]. Dassonville et al. (1997) found a greater volume of activation in the contra lateral motor cortex when the dominant hand was used, and moreover, a relation between the degree of handedness and the extent of lateralization [33].

The study’s very large number of subjects included 1980 children who preferred their right hand. The results provide more evidence to support the 10% rule, as it shows statistical differences in favor of the preferred hand in all age groups for RP children, and that this percentage is present from an early age and remains remarkably constant for both genders. Our study also included a relatively large number of children who preferred their left hand compared with other studies on this subject in children [8,9,13,23]; this enabled us to draw some conclusions about the minority of LP children and to examine differences between LP boys and girls in more detail. In contrast there are several limitations. First, this study is cross-sectional and therefore provides results that reflect a snapshot at a certain time. Children were not followed from age 4 to 14+ onwards, instead we choose a large group of children of a certain age. Second, dominance was not additionally confirmed by teachers or parents, hence we choose the term preference when referring to the current study. Third, the number of participating LP children aged >14 was relatively low. We attempted to compensate for this by pooling children aged 14 years or older into a single group for the statistical analyses. Finally, an observer bias cannot be ruled out, owing to the fact that this parameter wasn’t recorded in the database. In this context, the Jamar^®^ hydraulic hand dynamometer (JHD) and similar dynamometers have proven to have a high test–retest and inter-investigator reliability, as well as a high reproducibility when used by children [34–36].

Overall it can be concluded that the results of this study show that the 10% rule holds true for RP children, for both boys and girls in all age groups separately, but not for left-preferent children. LP girls are significantly stronger with their preferred hand, but this difference only amounts to 3%. For LP boys no difference in favor of either hand was found. LP children more often score higher with their non-dominant hand or tie scores on both hands than RP children. However, a small portion of RP children also display this effect.

## Supporting Information

S1 Appendix Score of the preferred hand of RP versus that of LP children and score of the non-preferred hand of RP versus LP children. (DOC)

S1 Database (SAV)
