# Supplementary material for: The Influence of Hand Preference on Grip Strength in Children and Adolescents; A Cross-Sectional Study of 2284 Children and Adolescents
Source: PLoS One. 2015 Nov 23;10(11):e0143476. doi: 10.1371/journal.pone.0143476 (PMC4657904; doi:10.1371/journal.pone.0143476)
Supplement: S1 Appendix — (DOC) [file pone.0143476.s001.doc]

**S1.**Appendix

Score of the preferred hand of RP versus that of LP children and score of the non-preferred hand of RP versus LP children.

|  | **Preferred hand** | | | **Non-preferred hand** | | |
| --- | --- | --- | --- | --- | --- | --- |
|  | **Mean Difference** | **SE*** | **P-value** | **Mean Difference** | **SE*** | **P-value** |
| **Total Group** | -1.614 | 0.494 | 0.001 | -0.271 | 0.494 | 0.583 |
| **Boys** | -2.763 | 0.687 | <0.001 | -1.206 | 0.687 | 0.079 |
| **Girls** | -0.498 | 0.714 | 0.486 | 0.535 | 0.714 | 0.454 |
| **Boys 4 yrs** | 0.421 | 0.768 | 0.584 | -0.443 | 0.768 | 0.564 |
| **Boys 5 yrs** | 1.242 | 0.981 | 0.206 | -0.065 | 0.981 | 0.947 |
| **Boys 6 yrs** | -0.398 | 0.905 | 0.660 | -0.807 | 0.905 | 0.373 |
| **Boys7 yrs** | 0.534 | 1.055 | 0.613 | -0.896 | 1.055 | 0.395 |
| **Boys 8 yrs** | 1.657 | 1.114 | 0.137 | 0.411 | 1.114 | 0.712 |
| **Boys 9 yrs** | 1.126 | 1.114 | 0.312 | -1.198 | 1.114 | 0.282 |
| **Boys 10 yrs** | -0.112 | 0.919 | 0.903 | -1.847 | 0.919 | 0.044 |
| **Boys 11 yrs** | 2.835 | 0.994 | 0.004 | 0.736 | 0.994 | 0.459 |
| **Boys 12 yrs** | 2.031 | 1.035 | 0.050 | -0.044 | 1.035 | 0.966 |
| **Boys 13 yrs** | 2.561 | 1.758 | 0.145 | 0.315 | 1.758 | 0.858 |
| **Boys 14^+^ yrs** | 1.777 | 1.428 | 0.214 | -2.741 | 1.428 | 0.055 |
| **Girls 4 yrs** | -1.330 | 1.117 | 0.234 | -1.529 | 1.117 | 0.171 |
| **Girls 5 yrs** | 0.539 | 1.256 | 0.668 | 0.076 | 1.256 | 0.952 |
| **Girls 6 yrs** | 1.067 | 1.051 | 0.310 | 0.470 | 1.051 | 0.655 |
| **Girls 7 yrs** | -0.481 | 1.181 | 0.684 | -1.102 | 1.181 | 0.351 |
| **Girls 8 yrs** | 0.153 | 1.195 | 0.898 | -1.033 | 1.195 | 0.388 |
| **Girls 9 yrs** | 1.238 | 1.075 | 0.250 | 0.314 | 1.075 | 0.770 |
| **Girls 10 yrs** | -0.082 | 1.055 | 0.938 | -1.658 | 1.055 | 0.116 |
| **Girls 11 yrs** | -0.827 | 1.048 | 0.430 | -2.109 | 1.048 | 0.044 |
| **Girls12 yrs** | 2.754 | 1.317 | 0.037 | -0.063 | 1.317 | 0.962 |
| **Girls 13 yrs** | 3.527 | 1.735 | 0.042 | 1.482 | 1.735 | 0.393 |
| **Girls 14^+^ yrs** | -4.300 | 1.280 | 0.001 | -4.725 | 1.280 | <0.001 |
